# Terrain Modeling and Cost Map Construction for Autonomous Agricultural Vehicles in Hilly Orchards: A Review

**DOI:** 10.3390/s26123793

**Published:** 2026-06-14

**Authors:** Ruohan Shi, Hanquan Lei, Yunfei Wang, Mingxiong Ou, Weidong Jia

**Affiliations:** 1 School of Agricultural Engineering, Jiangsu University, Zhenjiang 212013, China; 2222416009@stmail.ujs.edu.cn (R.S.); 2222516027@stmail.ujs.edu.cn (H.L.); 2112416035@stmail.ujs.edu.cn (Y.W.); myomx@ujs.edu.cn (M.O.); 2 High-Tech Key Laboratory of Agricultural Equipment and Intelligence of Jiangsu Province, Jiangsu University, Zhenjiang 212013, China

**Keywords:** hilly orchards, digital elevation model, terrain modeling, traversability assessment, cost map, autonomous navigation

## Abstract

Navigating hilly orchards is challenging for autonomous agricultural vehicles due to the rugged terrain and dense canopy cover. Standard environmental modeling techniques are widely used, yet they often overlook how elevation uncertainty propagates during Digital Elevation Model (DEM) reconstruction. This oversight can directly affect terrain risk assessments and navigation planning. From an error-propagation perspective, this review examines how uncertainties originating from RTK-GNSS, LiDAR, and computer vision propagate through DEM reconstruction, terrain-feature extraction, cost map construction, and path planning. We further analyze how DEM elevation errors and vertical inaccuracies affect slope estimation, roughness representation, traversability assessment, vehicle stability, and navigation safety. Finally, we highlight practical bottlenecks in hilly orchard scenarios and suggest several research priorities, including multimodal fusion, uncertainty-aware modeling, lifelong map updating, and learning-based traversability assessment.

## 1. Introduction

Modern orchard management is increasingly advancing toward mechanization [1], intelligence [2,3], and autonomy. This trend has increased the demand for autonomous agricultural vehicles capable of performing tasks such as soil cultivation, canopy management, pest control, irrigation [4,5], and harvesting. Compared with flat farmland, hilly orchards feature pronounced terrain variation [6], limited operating space, irregular tree distribution, and dense canopy cover. Navigating these unstructured environments is therefore challenging and imposes stringent requirements on system safety.

Environmental modeling for hilly orchard navigation should extend beyond obstacle detection. It requires an accurate representation of the 3D terrain surface because terrain geometry directly affects vehicle traversability, stability, and operational safety. Digital Elevation Models (DEMs) play a central role in this process. In addition to supporting topographic mapping and visualization [7], DEMs provide the basis for deriving key terrain features such as slope, aspect, curvature, roughness, and relief. DEM quality therefore affects not only terrain representation, but also the reliability of subsequent traversability assessment, risk evaluation, cost map construction, and path planning.

However, constructing reliable DEMs in hilly orchards remains difficult. The reconstruction process is vulnerable to GNSS degradation, point cloud sparsity, vegetation occlusion, changing illumination, and large local terrain variation [8]. These initial sensing errors do not remain isolated. GNSS vertical drift, LiDAR blind spots, vision-based depth errors, and ground-point extraction errors may be incorporated into DEM reconstruction and interpolation, producing elevation bias or local surface distortion. Once embedded in the DEM, these errors may distort derived terrain features such as slope, roughness, curvature, and breaklines. The distorted features may then propagate into traversability assessment and cost map construction, increasing the risk of unsafe or biased planning decisions.

**Figure 1:**
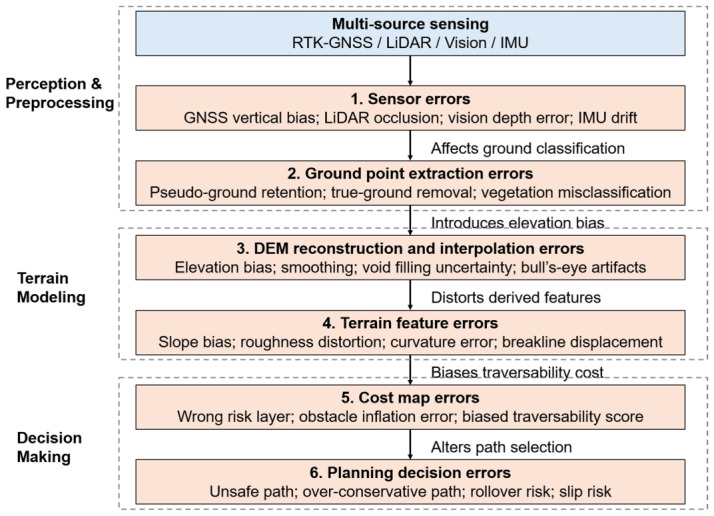
Error propagation chain in navigation-oriented terrain modeling for hilly orchards, showing how sensor errors propagate through ground point extraction, DEM reconstruction and interpolation, terrain feature derivation, cost map construction, and final planning decisions.

Although many studies have examined individual sensors, mapping accuracy, or local planning algorithms, fewer reviews systematically analyze the complete workflow from sensing uncertainty and DEM reconstruction to terrain-risk modeling, cost map construction, and navigation decisions. Previous reviews have discussed orchard phenotyping [9], general perception, and navigation methods, but DEM reliability in hilly orchards and its safety implications for autonomous navigation have received less attention. To clarify this review logic, Figure 1 summarizes the navigation-oriented error-propagation chain from sensing uncertainty to DEM reconstruction, terrain-feature derivation, cost map construction, and planning decisions.

To address these gaps, this review adopts error propagation as the central organizing thread. The sensing–DEM–terrain representation–cost map–navigation chain is used here as a review-based analytical structure, rather than as a standardized framework already widely adopted in hilly orchard navigation. This review first outlines the environmental and operational constraints of hilly orchards, then examines multimodal sensing, DEM reconstruction, terrain-feature extraction, cost map construction, and the effects of these processes on navigation decisions, before discussing emerging research directions for safer autonomous navigation.

To ensure the rigor of this comparative analysis, the literature discussed herein was primarily retrieved from major scientific databases (e.g., Web of Science, Scopus and Google Scholar), focusing on peer-reviewed studies published mainly between 2020 and 2026. The search keywords covered hilly orchards, autonomous agricultural vehicles, GNSS, LiDAR, vision-based perception, DEM reconstruction, traversability assessment, cost map construction, and path planning. Studies were included when they addressed field-applicable sensing, DEM generation, terrain-risk modeling, cost-map construction, or autonomous navigation, whereas studies unrelated to ground-vehicle traversability or navigation-oriented terrain modeling were excluded.

## 2. Environmental Characteristics and Operational Constraints in Hilly Orchards

Hilly orchards are representative unstructured agricultural environments [10]. Compared with conventional flat farmland, they exhibit more complex spatial layouts, stronger terrain variation, irregular tree distribution, and denser environmental elements [11]. Navigation in these environments must account for terrain constraints, tree-structure constraints, and vehicle operational limits. These physical constraints ultimately need to be converted into traversability indicators and cost-map layers to support path planning and motion control.

### 2.1. Terrain Constraints

Marked elevation changes and fragmented cultivable plots dominate hilly and mountainous environments [12]. Vehicles often encounter steep inclines, scattered ground obstacles, and poor surface conditions [13], indicating that 3D terrain geometry strongly influences operational safety.

Slope is one of the key geometric descriptors of terrain [14,15]. Unlike flat farmland, hilly orchards often contain uneven ground with typical inclines ranging from 5° to 15° [16]. Longitudinal slopes mainly affect climbing capacity, traction demand, and wheel-slip risk, whereas lateral slopes directly threaten roll stability and increase rollover risk [17]. Because orchard plots are often irregular, longitudinal and lateral slopes may occur together, creating compound slope conditions that continuously alter vehicle posture and stability.

Beyond general inclines, physical surface roughness introduces localized elevation variations that can induce repeated pitch and roll motions during vehicle movement [18]. These motions affect sensor stability and reduce the operational precision of attached tools such as sprayers or harvesting platforms [19]. It is important to distinguish physical terrain roughness from DEM-derived roughness metrics: the former increases ground sampling and reconstruction difficulty, whereas the latter is calculated from DEM elevations and is sensitive to elevation noise during feature extraction.

Discontinuous terrain features, such as ditches and steps, are also widespread in hilly orchards. Formed by natural erosion, manual land preparation, or road borders, these structures vary widely in height and width. If the size of a discontinuity exceeds the vehicle’s ground clearance or obstacle-crossing capability, the corresponding path may become impassable. Terrain modeling must capture abrupt structural changes and their spatial distribution, rather than relying solely on continuous slope data.

Taken together, hilly orchard navigation is constrained by slope, roughness, discontinuous obstacles, corridor width, and soil-state conditions. These constraints must be transformed into quantifiable traversability indicators before they can be used by path planners. Table 1 summarizes their corresponding indicators, operational risks, and cost-map layers.

### 2.2. Tree Structure Constraints

In addition to terrain variation, the spatial layout and structural characteristics of fruit trees impose important constraints on autonomous navigation in hilly orchards [20]. Most orchards are planted in rows, but rugged terrain often results in uneven row spacing, irregular plant distribution, and highly variable canopy structures. These factors not only restrict vehicle mobility but also affect environmental perception and map construction.

Canopy occlusion [21] can block or reflect GNSS signals [22], limit the sensing range of LiDAR and cameras, and leave parts of the environment only partially observed. In environmental models, canopy occlusion can be represented as a perception-confidence or uncertainty layer; regions with low observation confidence may be assigned higher traversability costs or require additional sensing before path execution.

Inter-row corridors define the effective navigable space for agricultural machinery. Their width is determined by trunk spacing, canopy spread, pruning conditions, and local terrain layout [23]. Corridor boundaries, row centerlines, and local corridor width can be extracted from trunk positions, canopy contours, or semantic segmentation results. These representations support traversable corridor extraction, define the feasible navigation envelope, and constrain the search space of path planners.

Canopy height, low branches, trunks, trellis structures, and overhanging vegetation impose additional clearance and collision constraints, especially for sprayers and harvesting robots [24]. These structures can be represented using vertical clearance maps, semantic obstacle layers, occupancy-grid cells, and obstacle inflation zones. Recent semantic mapping frameworks further show that orchard objects, such as trunks, canopy regions, trellis structures, and ground surfaces, can be assigned semantic labels and integrated into navigation-oriented map layers.

Overall, tree-structure constraints should be represented not only as discrete obstacles, but also as semantic, clearance, uncertainty, and corridor layers. Errors in detecting trunks, canopy boundaries, low branches, or corridor width may propagate into obstacle inflation, clearance estimation, feasible-corridor extraction, and final path selection. Therefore, accurately converting tree-structure characteristics into model-based constraints is essential for reducing cost-map errors and improving navigation safety in hilly orchards.

### 2.3. Vehicle Operational Constraints

Traversability depends not only on the environment but also on vehicle dynamics and operational limits. Rollover is one of the main safety risks during operation on sloped terrain. When a vehicle travels across lateral slopes or turns on uneven ground, the relationship between the center of gravity (CG) and the support polygon changes continuously. Once the CG projection moves outside the support region, the vehicle can lose stability [25].

A vehicle’s minimum turning radius also constrains route feasibility in narrow orchard corridors [26,27]. Curved rows, local corridor narrowing, and scattered obstacles may make a geometrically valid path difficult to execute under real field conditions.

Specific farming tasks, such as spraying and harvesting, require relatively stable vehicle posture [28,29]. Excessive pitch or roll reduces operational accuracy and may damage attached equipment [30]. Therefore, path planning should consider ride smoothness, posture stability, and task-specific operational constraints in addition to collision avoidance. Incorporating terrain-adaptive vehicle models [31] can support cost maps that better reflect these operational risks.

## 3. Terrain Perception and 3D Environment Construction in Hilly Orchards

Environmental perception provides the basis for autonomous agricultural operations in complex field environments [32]. In hilly orchards, perception involves more than identifying trees, paths, or obstacles. Reliable elevation information is equally important because terrain variation directly affects navigation safety and vehicle stability.

Sensors used for orchard terrain mapping are generally divided into two categories: absolute positioning sensors and relative perception sensors. GNSS is the most widely used absolute positioning method, while LiDAR and cameras are commonly adopted for relative environmental perception [33].

### 3.1. GNSS-Based Elevation Measurement and Positioning

In hilly orchard mapping, GNSS provides the absolute positioning reference required for georeferencing sensor trajectories, ground control points, UAV imagery, and mobile LiDAR point clouds. RTK-GNSS is widely used as an absolute reference for orchard terrain mapping [34]. For DEM construction, its main relevance lies in controlling absolute elevation consistency and reducing trajectory-level vertical offsets during point cloud registration and surface reconstruction.

GNSS degradation in hilly orchards is not caused by canopy blockage alone. Dense canopy, uneven terrain, and terrain shielding can reduce satellite visibility and signal strength. However, the number of visible satellites is not sufficient to characterize GNSS reliability. Satellite spatial distribution, commonly quantified by GDOP or PDOP, also strongly affects positioning accuracy. Therefore, even when enough satellites are visible, poor satellite geometry may still degrade positioning reliability and amplify the effects of canopy blockage and multipath interference.

Multipath interference is another important source of GNSS uncertainty. In addition to canopy reflections, orchards contain many reflective surfaces, including plastic mulch, irrigation infrastructure, metallic trellis systems, agricultural machinery, and protective nets or covers. These surfaces may introduce biased observations, especially when receivers operate near the ground or under partially occluded sky conditions. In DEM generation, multipath-induced vertical biases may be embedded into GCPs, UAV camera positions, LiDAR trajectories, ground-vehicle mapping paths, or point cloud registration, leading to elevation offsets, local geometric inconsistency, strip misalignment, and distorted terrain features.

Reliable GNSS-based positioning remains important for agricultural vehicle guidance and autonomous navigation [35]. Dynamic testing of low-cost multi-frequency and multi-GNSS receivers has shown their potential to improve positioning availability, RTK re-acquisition, and navigation performance in agricultural environments [36]. Comparative studies of GNSS positioning concepts further indicate that correction-service selection affects positioning stability and applicability in precision agriculture [37]. Satellite augmentation systems, such as QZSS, can improve navigation reliability for agricultural machinery under partially obstructed conditions [38]. Robust estimation methods, including epoch differencing, ambiguity validation, Kalman filtering, and factor graph optimization, can improve solution continuity and suppress outliers caused by satellite loss, cycle slips, or temporary signal degradation [39,40,41]. For DEM reconstruction, the value of these methods lies not only in positioning accuracy, but also in maintaining vertical trajectory consistency and reducing elevation jumps, strip misalignment, and local surface deformation.

Several correction and augmentation strategies are relevant to orchard DEM generation. SBAS services, such as WAAS, EGNOS, MSAS, and other regional augmentation systems, can improve positioning availability but are usually insufficient for high-resolution DEMs. Local RTK can provide centimeter-level positioning under favorable conditions, although it depends on baseline length, correction-link stability, and signal continuity. CORS-based network RTK supports regional operations without local base-station deployment, but it remains affected by local canopy occlusion and satellite geometry. PPP enables precise positioning without nearby base stations, but its convergence time and sensitivity to signal interruption limit real-time DEM use. PPP-RTK improves convergence through regional augmentation. PPK is particularly important for UAV-based mapping and DEM generation because it improves camera or LiDAR trajectory accuracy during post-processing without requiring a continuous real-time link, thereby reducing strip misalignment and improving DEM vertical accuracy and slope consistency.

Overall, GNSS evaluation for hilly orchard DEM construction should consider not only horizontal positioning accuracy, but also vertical accuracy, canopy robustness, satellite geometry, correction-link continuity, orchard applicability, and downstream relevance to DEM reconstruction. Representative GNSS correction, augmentation, and optimization strategies are summarized in Table 2.

### 3.2. LiDAR-Based 3D Point Cloud Mapping

LiDAR systems generate 3D point clouds by emitting laser pulses and recording the returned signals [50]. Because the measurements are directly related to spatial distance, LiDAR can describe terrain geometry with relatively high accuracy. In hilly orchards, it is widely used to capture terrain undulations, road edges, obstacle shapes, and abrupt elevation changes. LiDAR has become one of the main data sources for DEM reconstruction [51]. Depending on the platform, agricultural LiDAR breaks down into airborne (ALS), terrestrial (TLS), and mobile (MLS) scanning.

ALS is commonly mounted on UAVs and provides rapid top-down data acquisition over large areas [52]. It is useful for regional terrain surveying, slope analysis, orchard undulation assessment, and road-network extraction. However, under dense canopy, laser pulses may strike vegetation before reaching the ground, causing the reconstructed surface to resemble a DSM rather than a bare-earth DEM. Therefore, ALS is efficient for regional mapping but may have limited under-canopy ground penetration. Recent UAV-LiDAR studies have demonstrated its value for regional agricultural mapping, trajectory optimization, and 3D environmental modeling [53,54].

TLS usually operates from fixed stations and can generate very dense local point clouds [55]. Its close-range measurement mode is suitable for reconstructing detailed terrain structures such as road shoulders, stepped ditches, embankments, and tree trunks. However, TLS has low field efficiency and is sensitive to station occlusion, which limits its use for continuous route-level mapping.

**Figure 2:**
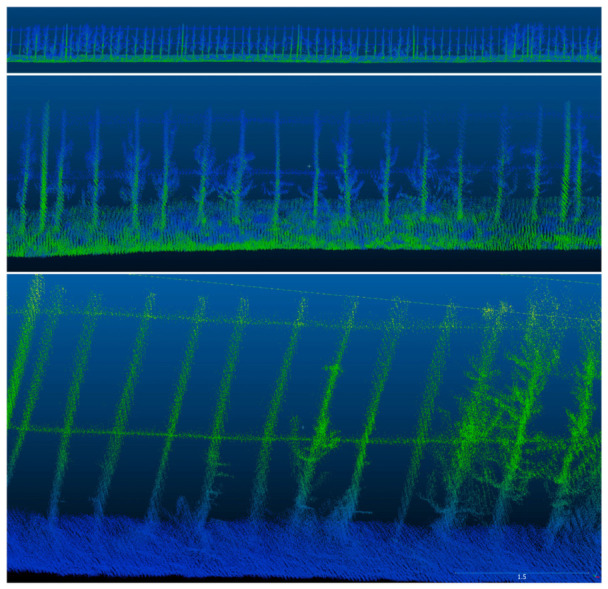
Reconstructed digital twin of an orchard row generated using Spatial Field-LOAM; top: complete row (around 65 m), middle: trees between two pillars, bottom: close-up, depicting tree trunks. This figure demonstrates the capability of Spatial Field-LOAM to maintain row-level reconstruction continuity, preserve local tree-trunk geometry, and support navigation-oriented orchard mapping under mobile LiDAR scanning conditions. Reproduced from Ref. [60] with permission.

MLS integrates LiDAR with positioning and orientation sensors on tractors, ground robots, or backpack platforms. Compared with TLS, MLS improves operational efficiency while retaining close-range terrain representation, making it suitable for route-level DEM construction and navigation support [56]. However, MLS performance depends strongly on time synchronization, attitude estimation, motion compensation, and scan registration. When the platform moves across rough or sloped terrain, motion distortion, swath misalignment, and local stretching may reduce DEM smoothness and bias local slope estimation. Recent MLS studies further show that LiDAR-SLAM [57], LiDAR–IMU fusion, scan matching, and adaptive map updating can support localization and mapping in GNSS-degraded agricultural environments [58,59]. Figure 2 presents an example of reconstruction results generated by Spatial Field-LOAM.

A horizontal comparison of ALS, TLS, and MLS is provided in Table 3. ALS is suitable for rapid regional terrain surveying but may have limited under-canopy ground penetration. TLS provides highly dense local point clouds for detailed terrain validation but has low field efficiency. MLS offers a compromise between scanning efficiency and close-range terrain representation, making it more suitable for route-level DEM construction and real-time navigation support.

From the perspective of error propagation, LiDAR measurement noise, vegetation occlusion, insufficient ground returns, motion distortion, and registration errors may first produce sparse or misaligned point clouds. These errors can then propagate into DEM surface distortion, artificial roughness, breakline loss, and biased slope estimation, ultimately affecting cost-map construction and navigation decisions.

### 3.3. Vision-Based Semantic Mapping

Vision sensors are widely used in orchard environmental perception because of their low cost, flexible deployment, and ability to provide both geometric and semantic information [61,62]. In DEM-related applications, vision-based methods include active depth sensing, such as structured light and Time-of-Flight (ToF), and passive reconstruction, such as stereo vision and Structure from Motion (SfM). Active methods can provide local depth information but are affected by illumination and sensing range, whereas passive methods reconstruct 3D structures from image disparity or multi-view feature matching but are sensitive to texture quality, occlusion, and camera pose estimation [63,64]. Semantic segmentation, object detection, and ground recognition further support DEM preprocessing by distinguishing ground surfaces from trees, weeds, roads, ditches, and artificial structures [65,66].

Deep learning has improved visual perception under complex agricultural conditions, especially for ground segmentation and monocular depth estimation [67,68,69]. However, these models usually require large annotated datasets, and their cross-orchard or cross-season generalization remains uncertain. Therefore, although vision can support local terrain reconstruction and semantic mapping, it rarely produces a stable bare-earth DEM alone under dense-canopy conditions and often requires LiDAR, GNSS/IMU, or prior map information for reliable DEM construction [70].

Vision-based DEM reconstruction and semantic mapping remain strongly scenario-dependent. Lighting variation changes image contrast and shadow distribution, reducing feature matching, stereo depth estimation, and segmentation consistency. Repetitive textures in tree rows, canopy regions, or bare soil may cause mismatched features and unreliable SfM camera poses. Canopy occlusion blocks ground visibility, resulting in incomplete bare-earth reconstruction and fragmented traversable-region extraction. Low-texture surfaces, such as bare soil, muddy paths, or homogeneous grass, provide insufficient features for stable depth recovery. Seasonal changes further alter canopy density, illumination, ground cover, and object appearance, reducing the transferability of segmentation and depth-estimation models. An overview of representative vision-based reconstruction and semantic mapping studies is summarized in Table 4, with emphasis on data type, experimental scenario, semantic output capability, scenario-dependent limitations, and DEM applicability.

From the perspective of error propagation, camera calibration errors, lighting variation, texture deficiency, and canopy occlusion may cause depth errors, incomplete ground reconstruction, or semantic misclassification. These errors can propagate into DEM surfaces, terrain-feature extraction, cost-map layers, and final navigation decisions.

### 3.4. Multimodal Fusion Mapping

Hilly orchards present combined challenges, including uneven terrain, dense canopy cover, narrow operating corridors, and changing local environments. Under these conditions, relying on a single sensor is often insufficient. Elevation accuracy, terrain-continuity representation, robustness to occlusion, and real-time performance are difficult to maintain simultaneously. Therefore, multimodal fusion has become an important direction for orchard mapping and navigation [77].

Most multi-sensor fusion frameworks can be understood through three functional layers: positioning-level fusion, local geometric reconstruction, and semantic enhancement. At the positioning level, GNSS and inertial measurement units (IMUs) are commonly integrated to establish a stable pose reference. GNSS provides global positioning constraints, while INS dead reckoning maintains short-term pose continuity during temporary signal degradation. However, in hilly orchards, canopy occlusion, terrain shielding, and multipath interference may still degrade GNSS reliability, making additional LiDAR or vision-based constraints necessary.

At the local geometric reconstruction level, LiDAR–IMU fusion is widely used to maintain continuous odometry and point cloud registration in GNSS-degraded environments. Tightly coupled LiDAR/IMU localization and mapping methods have been developed for orchard robots to improve real-time mapping robustness [78]. LiDAR-SLAM-based orchard inspection robots and LiDAR–IMU operation-platform mapping further demonstrate the value of close-range geometric reconstruction for agricultural navigation [79,80]. Additional orchard SLAM studies incorporating trunk features, loop closure optimization, LIO-SAM, AprilTag constraints, and multi-sensor fusion have been used to improve localization continuity and terrain mapping consistency [81,82,83]. To reduce accumulated drift during longer operations, 3D SLAM and point-cloud positioning methods have also been combined for orchard navigation [84]. Other studies on agricultural machinery localization, LiDAR–IMU SLAM, and LiDAR-based orchard navigation further indicate the importance of robust mapping and localization in structured agricultural environments [85,86,87]. Efficient LiDAR–visual–inertial odometry has been applied to agricultural unmanned ground vehicles to improve continuous localization and local mapping accuracy [88], while tightly coupled GNSS/LiDAR/IMU fusion has been used to improve orchard mapping robustness under GNSS degradation [89]. These fusion strategies improve point cloud registration and support more reliable separation of ground and non-ground points during DEM, DSM, and canopy height model construction.

At the semantic enhancement level, visual sensors provide texture, color, object-level information, and semantic labels that are difficult to obtain from LiDAR geometry alone. Vision-based mapping methods, including UAV remote sensing, object-detection-based perception, and visual SLAM–inertial fusion, indicate the potential of visual information for dense scene reconstruction and map refinement [90,91,92]. LiDAR–camera fusion can support visual navigation, obstacle avoidance, and semantic understanding for agricultural robots [93]. GNSS–vision integration has also been applied to autonomous navigation and scene modeling for trellis orchard transportation robots, improving localization and environmental representation in structured orchard corridors [94]. UAV-based multi-view imagery combined with GNSS/IMU can support large-scale semantic real-time mapping, although weak vegetation penetration still limits bare-earth DEM extraction under dense canopy [95].

Although multimodal fusion improves positioning robustness, geometric reconstruction, and semantic interpretation, it does not eliminate DEM uncertainty. Orchard floors are often covered with weeds, fallen leaves, low branches, and pseudo-ground objects. As a result, fused point clouds may still contain points that do not represent the true bare-earth surface. Therefore, high-quality DEM construction still requires uncertainty-aware ground extraction, trajectory consistency checking, and validation of DEM-derived terrain features before these maps are used for navigation decisions.

## 4. High-Precision DEM Reconstruction and Quality Assessment in Hilly Orchards

Collecting multimodal elevation data is only the first step; the main challenge lies in converting raw, noisy, and scattered point clouds into a continuous and reliable terrain surface. Raw measurements cannot be fed directly into navigation algorithms. Instead, they must undergo a structured reconstruction pipeline, including initial cleaning, interpolation, multi-scale representation, and feature extraction, to build a DEM that accurately represents orchard topography.

### 4.1. Data Preprocessing and Ground Point Extraction

Raw orchard datasets are often complex and heterogeneous. Point clouds are frequently affected by canopy occlusion, sensor noise, drift, and highly variable sampling densities. Because they contain large numbers of leaves, branches, and floating artifacts, direct use of these data for DEM generation is generally infeasible. Before any surface modeling begins, the data require filtering and ground-point extraction [96].

Basic preprocessing usually includes coordinate unification, outlier removal, and local gap filling. The main challenge, however, is separating the actual ground surface from low-hanging canopies and weed cover. Current extraction methods range from elevation thresholding, morphological filtering, progressive TIN densification, and grid-based filtering to geometric feature methods and learning-based segmentation. These methods differ substantially in filtering mechanism, sensitivity to vegetation, ability to preserve steep terrain structures, and applicability to navigation-oriented DEM reconstruction. To address the large volume of redundant data, Li et al. [97] designed an octree-based 3D point cloud optimization method. By adaptively partitioning space, their approach removes redundant points while preserving critical terrain and obstacle features required for navigation. Representative implementations include grid-based ground estimation [98], data-driven morphological filtering [99], and neural-geometric hybrid segmentation [100].

Ground extraction is particularly challenging in hilly orchards. Dense weeds may interfere with both LiDAR and camera perception, blurring the boundary between low vegetation and the actual soil surface. If weed points are not removed, the resulting DEM may be artificially elevated, generating false rough-terrain artifacts across the map. Conversely, overly aggressive filtering can also introduce errors. Distinct ditches, terrace steps, and sharp slope transitions may be removed or overly smoothed by excessive denoising. To address this issue, recent research has shifted from simple noise removal toward retaining critical topographic discontinuities, ensuring that the final DEM better supports field navigation. To clarify the suitability of different filtering strategies for hilly orchard DEM reconstruction, Table 5 compares common ground point filtering methods in terms of filtering effect, advantages, limitations, and applicable conditions.

### 4.2. DEM Generation and Interpolation Optimization

Once ground points have been extracted, spatial interpolation or surface fitting is required to generate a continuous DEM. This stage involves a trade-off between noise suppression, gap filling, and preservation of structural terrain features. In hilly orchards, terrain includes gradual slopes and abrupt discontinuities; navigation-oriented DEMs should preserve both continuity and critical features such as breaklines, ditches, ruts, and terraces.

Spatial interpolation is central to DEM generation. IDW is computationally efficient but may create local artifacts under sparse sampling; Spline produces smooth surfaces but may weaken sharp terrain transitions; Kriging accounts for spatial autocorrelation but depends on variogram selection; TIN-based methods better preserve discontinuities by adapting node density to terrain complexity; and learning-based filling can recover occluded elevations but requires representative training data and uncertainty control [101].

From an error-propagation perspective, interpolation artifacts are particularly important for navigation-oriented DEMs because they may be amplified during slope, roughness, and breakline extraction. For example, IDW may produce the well-known bull’s-eye effect [102], where artificial local highs or lows appear around sampled points under sparse or uneven point distributions. These artifacts may be misinterpreted as rough terrain, local obstacles, or discontinuities in cost maps. Spline interpolation may oversmooth sharp terrain transitions or generate overshoot near steep changes, leading to missed ditches or distorted slope estimates. Kriging may produce biased surfaces if the variogram model is poorly selected, while TINs may introduce triangular facet artifacts in sparsely sampled regions. Therefore, interpolation methods should be evaluated not only by elevation RMSE, but also by their effects on slope preservation, roughness stability, breakline continuity, obstacle-boundary consistency, and cost-map reliability.

The final DEM can be represented using either TINs or regular raster grids. TINs are advantageous for preserving abrupt terrain structures because node density can vary with local surface complexity, whereas regular grids are easier to integrate with grid-based path planners and cost maps. Many navigation-oriented workflows therefore adopt a hybrid strategy: terrain structure is first reconstructed or constrained using TINs or breakline-aware methods, and then converted into raster grids for efficient cost-map construction and path planning.

In addition to interpolation method selection, DEM resolution is a critical parameter that determines how elevation errors are transferred into terrain features and cost maps. An overly fine grid may represent residual sensor noise or interpolation artifacts as artificial micro-terrain, whereas an overly coarse grid may smooth out narrow ditches, ruts, steps, or terrace edges. A practical DEM resolution should be selected by jointly considering ground-point density, average point spacing, vehicle geometry, minimum obstacle size, interpolation uncertainty, computational constraints, planning frequency, and task requirements. If the grid size is smaller than the reliable observation spacing, DEM-derived slope and roughness may mainly reflect noise or interpolation artifacts. If it is larger than the characteristic scale of ditches, ruts, steps, or terrace edges, critical non-traversable features may be smoothed out. Therefore, multi-resolution or hierarchical DEMs may be more appropriate than a single fixed-resolution grid for hilly orchard navigation. Previous studies have also shown that DEM interpolation and grid-scale selection can significantly affect DEM-derived roughness estimates [103].

To better reflect navigation-oriented DEM requirements, typical interpolation artifacts and their potential downstream effects are also summarized in Table 6.

Overall, DEM generation is the key intermediate stage where upstream sensing and ground-point extraction errors are converted into terrain-representation errors. Interpolation artifacts, inappropriate grid resolution, and excessive smoothing may alter elevation, slope, roughness, curvature, and breakline information, thereby reducing the reliability of subsequent terrain-feature extraction.

### 4.3. Representation of DEM-Derived Terrain Features

Once a DEM is generated, terrain features relevant to vehicle mobility must be extracted and organized into planner-usable representations [107]. Slope and aspect are basic DEM-derived metrics and are commonly calculated using local moving-window methods, such as the Horn or Zevenbergen–Thorne algorithms. For navigation safety, slope should be evaluated relative to vehicle heading. Longitudinal slope mainly affects climbing resistance and slip risk, whereas lateral slope is closely related to rollover stability. Aspect further describes slope orientation and can support traction and drainage-related interpretation.

Surface roughness is another important DEM-derived attribute. It is commonly quantified using local elevation variation, height residuals, or 3D-to-2D area ratios. For agricultural tasks such as spraying and fruit harvesting, roughness is relevant not only to traversability, but also to chassis vibration, sensor stability, and implement operation accuracy. However, because DEM-derived roughness is calculated from elevation values, it is sensitive to elevation noise, interpolation artifacts, and grid resolution.

Discontinuous structures, such as embankments, ditches, ruts, terrace edges, and sharp breaklines, should also be explicitly represented. Unlike average slope or roughness values, these localized features may act as discrete physical barriers and directly determine whether a terrain patch is traversable. Preserving and identifying such abrupt terrain changes is therefore essential for evaluating the practical applicability of navigation-oriented DEMs.

### 4.4. Navigation-Oriented DEM Quality Assessment and Error Propagation

Evaluating DEM quality requires more than basic geometric metrics such as RMSE, mean bias, or absolute error. Although these indicators describe geometric agreement with reference data, they do not necessarily indicate whether the DEM is reliable for agricultural vehicle navigation. Small elevation errors may be amplified when DEM-derived features such as slope, roughness, curvature, and hazard boundaries are calculated.

From an error-propagation perspective, DEM inaccuracies may trigger a cascading error-transmission process. Raw observational errors, such as GNSS vertical drift, LiDAR occlusion, or point cloud sparsity, can first affect ground-point extraction. These errors may then be embedded into the reconstructed DEM during interpolation or surface fitting. Once incorporated into the DEM, they can distort terrain features and subsequently bias traversability assessment, cost-map layers, and path-planning decisions. As a result, non-traversable regions may be missed, risk costs may be underestimated or overestimated, and planners may generate unsafe or overly conservative paths.

Therefore, based on the reviewed literature, this review interprets orchard DEM evaluation from a navigation-oriented perspective that links geometric accuracy, terrain-risk consistency, and planning-level effects. This perspective is used as a synthesis for connecting DEM quality assessment with downstream navigation safety, rather than as a fixed evaluation standard. At the geometric level, elevation accuracy, surface continuity, and structural feature preservation are assessed. At the risk level, slope, roughness, discontinuities, and obstacle boundaries are examined in relation to physical terrain hazards. At the decision level, the resulting cost maps and planned trajectories are evaluated in terms of safety, stability, and executability for agricultural vehicles.

This multi-level interpretation helps clarify how DEM accuracy may influence downstream navigation decisions. In practice, DEM and navigation-related uncertainty can be quantified or represented in several forms, including elevation error statistics such as RMSE, MAE, bias, standard deviation, and confidence intervals; uncertainty rasters or cell-wise confidence layers derived from point density, interpolation variance, or sensor quality; pose covariance from GNSS/INS or SLAM systems; and probabilistic traversability or risk values in cost maps. Monte Carlo perturbation or repeated observations can further be used to examine how elevation or pose uncertainty affects slope, roughness, cost values, and path robustness. Table 7 summarizes representative studies on DEM quality assessment, error propagation, and navigation impacts.

## 5. Terrain Risk Modeling and Unified Cost Map Construction Methodologies

A geometrically accurate DEM is only the basis for autonomous navigation; the key task is to convert terrain information into computable risk and cost representations that can be used by path planners. Existing studies have considered different cost-related factors, including DEM-derived slope and roughness, terrain discontinuities, obstacles, semantic constraints, soil-state factors, and vehicle kinematics. However, a complete and standardized uncertainty-aware DEM–cost map–planning framework has not yet been widely established for hilly orchard navigation. Therefore, this section uses unified cost map construction as an organizing concept to compare existing approaches and to synthesize how heterogeneous environmental and operational constraints can be represented for path planning. This discussion includes three aspects: extracting risk factors from DEM-derived terrain features, incorporating non-topographic and vehicle-related constraints, and normalizing and fusing these factors into cost layers.

### 5.1. DEM-Based Risk Factor Modeling

Raw elevation values alone provide insufficient information for effective path planning. Instead, planners require terrain factors that can quantify traversability, stability, and operational risk. Earlier studies have provided theoretical and methodological foundations for classifying and filtering terrain-related risks [111].

In hilly orchards, major DEM-derived risk factors include longitudinal slope, lateral slope, surface roughness, terrain discontinuities, and abrupt elevation changes. Longitudinal slope is closely related to climbing resistance, traction demand, and wheel slip, whereas lateral slope is directly associated with rollover stability. Surface roughness reflects local elevation variation and is often related to chassis vibration, sensing instability, and task performance. Abrupt structural changes, such as ditches, steps, and breaklines, may represent obstacle-crossing risks or non-traversable barriers.

These terrain factors must be converted into cost values before they can be used by path planners. Several cost-conversion strategies are commonly used. Threshold-based classification converts terrain factors into discrete traversability classes, such as safe, risky, or non-traversable. For example, a slope or step height exceeding a predefined vehicle-specific threshold can be treated as a high-cost or impassable region. Continuous penalty functions assign gradually increasing costs as slope angle, roughness, or obstacle height increases, which helps avoid abrupt changes in path optimization. Fuzzy membership functions are useful for representing transitional risk states, for example when a slope is still physically traversable but may reduce vehicle stability. Probabilistic models represent risk as the likelihood of slip, rollover, or obstacle-crossing failure under uncertain terrain conditions. Learning-based cost functions can further infer traversability scores from DEM-derived features, vehicle response, historical driving data, or expert demonstrations. Therefore, DEM-based risk modeling should not only identify terrain features, but also define how these features are mathematically transformed into planner-usable cost values.

The choice of cost function affects navigation behavior. A strict threshold may generate conservative paths by excluding marginally traversable areas, while a continuous cost function can support smoother path optimization. However, if elevation noise or interpolation artifacts alter slope and roughness values, the resulting cost values may also be biased. From an error-propagation perspective, DEM-derived risk factors should therefore be evaluated together with their sensitivity to elevation uncertainty, resolution, and interpolation method.

**Figure 3:**
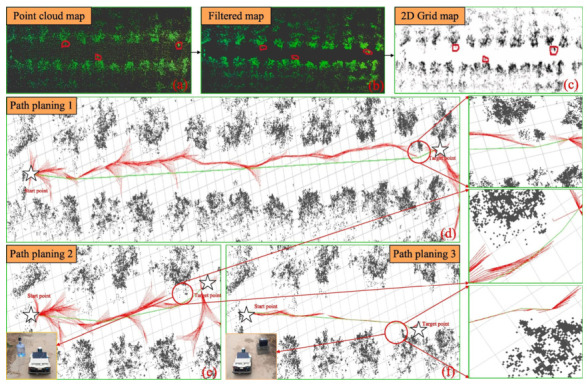
Path planning results for Scenario 3: (**a**) Prior 3D point cloud map of the orchard environment; (**b**) Point cloud map after ground filtering and SOR filtering, with obstacles preserved; (**c**) 2D grid map used for path planning; (**d**–**f**) Planned paths for the robot to bypass the bucket, pedestrian, and toolbox, respectively. The colored path curves indicate the planned robot trajectories generated using RRT*. This figure demonstrates how 3D point cloud mapping, filtering, and grid-map construction can be transformed into navigable paths using RRT* planning. Reproduced from Ref. [112] with permission.

In practice, researchers have integrated DEM-derived factors directly into navigation frameworks. Cao et al., for example, linked an improved LeGO-LOAM framework with RRT* algorithms and incorporated terrain risk information into trajectory generation [112]. Figure 3 illustrates how 3D point cloud mapping and traversability assessment can support safe path planning. Table 8 summarizes typical DEM-derived risk factors, possible cost representations, and their navigation meanings.

### 5.2. Non-Topographic Constraint Modeling

Topography alone does not determine whether an agricultural vehicle can safely traverse an orchard environment. Orchard navigation is also constrained by tree structures, spatial clearance, localization reliability, vehicle dynamics, and soil conditions. Therefore, a practical decision-making framework should quantify both topographic and non-topographic constraints and represent them as explicit cost or constraint layers [113,114].

Physical obstacles, such as trunks, low branches, trellises, rocks, and artificial structures, usually represent hard constraints. These obstacles can be modeled using occupancy grids, semantic obstacle layers, distance transforms, or obstacle inflation zones [115]. Even in the absence of a direct obstacle, narrow inter-row corridors, sagging branches, or localized canopy intrusion may reduce the effective navigable space and should therefore be represented as clearance or corridor-width constraints.

In agricultural environments, traversability also depends strongly on soil-state and vehicle–terrain interaction factors. Soil moisture can reduce bearing capacity after rainfall and increase sinkage or rut formation [116]. Low traction conditions may increase wheel slip, reduce path-tracking accuracy, and increase energy consumption [117]. Soil compaction, surface cover, weeds, fallen leaves, and loose soil may further alter the contact condition between the wheel and ground. Therefore, a geometrically smooth or low-slope area may still be unsafe after rainfall, while a steeper area may remain traversable when traction and bearing conditions are favorable. This means that orchard traversability should be regarded as a joint function of terrain geometry, soil mechanical state, surface condition, vehicle dynamics, and task requirements.

These non-geometric factors can be represented as additional cost layers. Soil moisture or bearing-capacity maps can be used to penalize areas with high sinkage risk. Slip-risk layers can be estimated from wheel odometry, IMU response, traction models, or historical vehicle behavior. Clearance maps can represent vertical constraints imposed by low branches and canopy height. Localization-confidence layers can penalize regions with severe canopy occlusion or GNSS degradation. Task-specific layers can encode operational requirements, such as stable posture for spraying, low vibration for harvesting, or high load-bearing capacity for transport.

**Figure 4:**
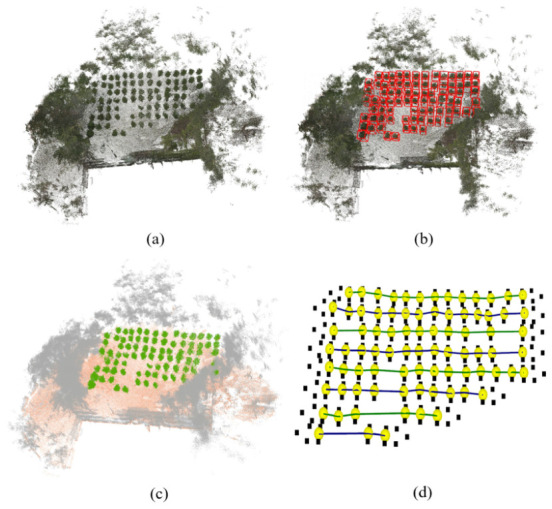
Semantic mapping and navigation graph construction in an orchard environment. (**a**) Original point cloud map; (**b**) object-level perception results generated by the 3D object detection network, showing detected fruit trees; (**c**) semantic mapping results after combining fruit-tree perception with terrain analysis, where the global map is classified into fruit trees, traversable terrain, and other obstacles; (**d**) visibility graph map constructed from tree-row structure and semantic information, providing high-level guidance for path planning and obstacle avoidance. This figure illustrates how raw point clouds can be transformed into semantic and graph-based navigation layers for structured orchard navigation. Reproduced from Ref. [118] with permission.

Beyond physical barriers, canopy structures also introduce perceptual blind spots and localization drift. These uncertainties can be represented using confidence-based layers or probabilistic penalties. To move beyond purely geometric modeling, recent frameworks have increasingly integrated semantic information. Semantic labels derived from LiDAR-SLAM or vision-based perception can distinguish trunks, canopy regions, ground surfaces, weeds, and obstacles, allowing different objects to be assigned different costs in the navigation map. Figure 4 shows how semantic mapping separates raw point clouds into categories that can be converted into navigation-oriented map layers.

### 5.3. Normalization and Fusion of Multimodal Cost Factors

Cost map construction requires heterogeneous variables, such as slope angle, roughness, obstacle distance, soil condition, and localization confidence, to be normalized into a comparable scale. Standard min–max scaling, piecewise functions, and threshold-based normalization are suitable for simple cases. Fuzzy logic is useful for representing gradual transitions between safe, risky, and non-traversable terrain. For example, a slope may still be physically traversable but may receive a high cost because it reduces stability or increases slip risk.

After normalization, different risk factors can be fused into a unified cost representation. The determination of fusion weights or fusion parameters should be guided by the dominant factors that most strongly affect navigation safety and task feasibility in a given scenario. For example, lateral slope and stability margin should be emphasized for rollover-prone vehicles operating on steep terrain, soil moisture and slip-related indicators should receive higher priority under low-traction conditions, while clearance, obstacle proximity, and localization confidence may dominate in narrow orchard corridors. For task-oriented operations such as spraying or harvesting, roughness, vibration, and posture stability may also require higher weights because they directly affect operation quality. Therefore, cost-map weights should not be regarded as universal constants, but as vehicle-, site-, and task-dependent parameters determined by safety limits, expert knowledge, field calibration, or vehicle-response data such as slip ratio, vibration, tracking error, and energy consumption. Common fusion strategies include linear weighted fusion, maximum-risk selection, fuzzy inference, Bayesian fusion, probabilistic risk modeling, and learning-based fusion. Linear weighted fusion is simple and interpretable, but it may mask extreme hazards when a high-risk factor is averaged with several low-risk factors. Maximum-risk selection avoids this problem by preserving the dominant risk, but it may generate overly conservative paths. Bayesian or probabilistic frameworks can represent uncertainty at the grid-cell level and update risk estimates as new observations become available. Learning-based methods can capture complex relationships among terrain, vehicle response, and task performance, but their generalization and interpretability remain challenging.

Real-world risks are often coupled rather than independent. A lateral slope may be moderately risky by itself, but the rollover risk can increase substantially when the vehicle turns sharply on that slope. Similarly, a narrow corridor may be traversable under accurate localization, but it becomes risky when close-range obstacles or GNSS degradation are present. Soil moisture may also interact with longitudinal slope by increasing wheel slip during climbing. These examples show that cost map construction should consider nonlinear coupling among terrain geometry, soil condition, obstacle proximity, localization confidence, vehicle dynamics, and task requirements.

From an error-propagation perspective, cost fusion is also the stage where upstream DEM and perception errors are transformed into navigation decisions. If slope, roughness, obstacle boundaries, or semantic labels are biased, the resulting cost layers may misrepresent traversability. Therefore, uncertainty-aware cost maps should preserve confidence information from sensing and DEM reconstruction, rather than fusing all layers into a deterministic cost value without uncertainty representation.

Current research is therefore moving from simple linear cost stacking toward fuzzy boundaries, probabilistic fusion, uncertainty-aware cost layers, and vehicle–terrain interaction models. Table 9 summarizes representative studies on the normalization and fusion of multimodal cost factors. However, in many existing studies, cost weights are still selected empirically or through limited field calibration, and their transferability across different orchard sites, soil conditions, and vehicle platforms remains insufficiently validated.

### 5.4. Unified Cost Map Construction

Early planners often used binary occupancy grids, in which each cell was classified as either free or occupied. Although computationally efficient, binary maps cannot represent continuous terrain variations such as slope, roughness, or vehicle kinematics. Because terrain fidelity significantly influences global path planning success, binary maps are insufficient for navigation in hilly orchards [125].

Modern approaches aim to integrate heterogeneous environmental and operational constraints into a unified, computable cost map [126,127]. Two main strategies have emerged: multi-layer 2D cost fusion and true 3D volumetric modeling.

In the multi-layer approach, different environmental datasets—including slope, roughness, physical obstacles [128,129], and kinematic constraints [130]—are represented as overlapping 2D cost layers, which are subsequently fused into a single navigable cost map. Zang et al. demonstrated that multi-layer architectures improve environmental context representation in obstacle-dense orchards [131]. However, these approaches incur increased computational requirements.

**Figure 5:**
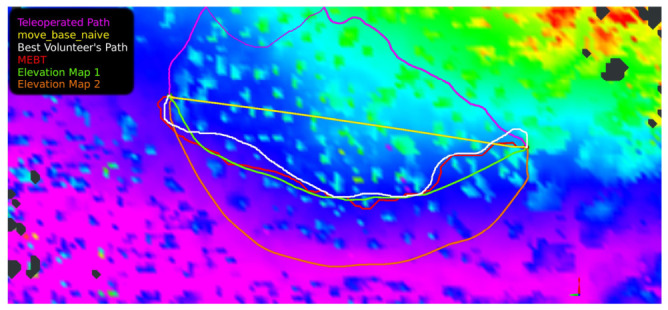
Comparison of paths generated by different terrain representation and cost modeling methods. The background elevation map uses a rainbow color scale, where magenta indicates lower terrain and the colors from blue, cyan, green, yellow, to red indicate increasing elevation. The robot icon represents the starting point, the flag represents the final goal, and the path lines indicate alternative traversing paths over the terrain. The figure illustrates how variations in terrain representation, traversability evaluation, and cost assignment can influence the final path selected by the planner. Reproduced from Ref. [135] with permission.

To address the limitations of 2.5D representations, some studies have developed 3D cost maps using voxels or surface meshes. Learning-based models can fuse geometric and visual data to assign traversability costs to discrete voxels [132]. Applying boundary and mesh generation to voxel-filtered point clouds supports robust path planning in complex terrains [133]. Surface meshes, as implemented in frameworks such as MeshNav3D, provide high-resolution navigation benchmarks for uneven ground [134]. Carvalho et al. integrated terrain slopes and vehicle load into 3D point cloud representations, linking spatial geometry directly to path planning [135]. Figure 5 illustrates how different terrain representation and cost modeling strategies can lead to different planned paths. This comparison highlights that path generation is not only determined by the planning algorithm itself, but also by how terrain slope, obstacle distribution, vehicle constraints, and traversability costs are represented in the map. According to Esfandiyar and Belter [136], voxel-surface mesh integration allows fine-grained cost representation but remains sensitive to data noise and requires careful weight tuning.

Deep learning approaches are increasingly applied to predict traversability costs from geometric, visual, or semantic data. Techniques such as synthetic dataset training, self-supervised learning, and unsupervised domain adaptation can generate voxel- or mesh-based cost predictions without exhaustive manual labeling [137]. These methods improve environmental modeling capability, although cross-scenario generalization and interpretability remain challenging. Overall, unified cost maps translate complex orchard terrain into structured planning representations, helping reduce the propagation of terrain-modeling errors into unsafe navigation decisions.

## 6. Current Challenges and Future Directions

### 6.1. Major Challenges

Although substantial progress has been made in terrain perception, DEM reconstruction, traversability assessment, and cost map construction, several challenges remain unresolved in hilly orchard environments. These challenges are summarized below according to sensing, DEM reconstruction, risk modeling, cost map representation, datasets, and long-term updating.

#### 6.1.1. Sensing Uncertainty

Reliable terrain modeling first depends on stable and accurate perception. As reviewed in Section 3, GNSS, LiDAR, and vision sensors each introduce uncertainty under hilly orchard conditions, including positioning instability, vegetation occlusion, motion distortion, and visual reconstruction errors. The remaining challenge is not only to improve sensor accuracy, but also to quantify sensing confidence and propagate it into ground-point extraction, point-cloud registration, DEM reconstruction, and downstream cost-map layers.

#### 6.1.2. DEM Reconstruction Uncertainty

As discussed in Section 4, DEM reconstruction uncertainty arises from ground filtering, interpolation, resolution selection, and structural feature preservation. The key challenge is to suppress noise and pseudo-ground points while retaining critical terrain discontinuities such as ditches, ruts, terrace edges, and breaklines. Future DEM reconstruction methods should therefore evaluate not only elevation accuracy, but also the stability of derived terrain features and their effects on traversability assessment and path feasibility.

#### 6.1.3. Risk Modeling Uncertainty

Translating terrain features into vehicle-level risk remains uncertain. Existing traversability models often rely on geometric indicators such as slope, roughness, and obstacles, but agricultural traversability is also affected by soil moisture, bearing capacity, wheel slip, traction conditions, sinkage, rut depth, vehicle load, and task requirements. A geometrically smooth surface may become unsafe after rainfall, while a steeper surface may remain traversable under favorable traction conditions. Therefore, terrain risk should not be evaluated using fixed geometric thresholds alone.

#### 6.1.4. Cost Map Representation

Cost maps form the interface between environmental modeling and path planning, but current representations remain limited. Binary occupancy grids cannot express continuous terrain risk, whereas DEM-based 2.5D maps may not fully represent vertical clearance, overhanging branches, or uncertainty. Multi-layer cost maps can integrate slope, roughness, obstacles, semantic constraints, soil-state factors, and vehicle kinematics, but their fusion weights are often manually tuned and scenario-dependent. Future cost maps should better incorporate uncertainty layers, nonlinear risk coupling, and vehicle–terrain interaction models.

#### 6.1.5. Datasets and Benchmarks

The development of robust terrain modeling and navigation algorithms is limited by the lack of public benchmark datasets for hilly orchards. Existing agricultural datasets often focus on flat farmland, crop rows, or general perception tasks, and rarely include steep slopes, dense canopy occlusion, GNSS degradation, narrow corridors, soil-state changes, and seasonal variation together. In addition, many studies evaluate individual modules using localization accuracy, point-cloud alignment, or DEM RMSE, while paying less attention to downstream effects on slope estimation, cost-map reliability, and path-planning safety. Future benchmarks should include multimodal sensing data, ground-truth elevation, soil-state measurements, vehicle response data, and navigation-level evaluation metrics.

#### 6.1.6. Long-Term Map Updating

Hilly orchards are dynamic environments, and one-time mapping is insufficient for long-term autonomous operation. Canopy growth, pruning, fallen branches, fruit load, and seasonal vegetation alter perception conditions and navigable space. Rainfall, machinery traffic, rut formation, soil compaction, erosion, sediment deposition, and local surface deformation can further change DEM geometry and traversability. Therefore, long-term orchard maps should update not only geometric obstacles, but also terrain-state layers such as soil moisture, rut depth, surface deformation, and traversability confidence.

### 6.2. Future Directions

Based on the reviewed literature and the limitations discussed above, future research should move from module-level optimization toward uncertainty-aware and navigation-oriented terrain modeling. First, multimodal sensing systems should improve DEM reconstruction while explicitly estimating uncertainty from sensing, ground-point extraction, interpolation, and georeferencing. Second, DEM evaluation should be linked with downstream navigation indicators, including slope classification accuracy, traversability consistency, rollover risk, and path feasibility, rather than relying only on elevation RMSE. Third, cost maps should integrate geometric, semantic, soil-state, and vehicle-dynamic constraints to better reflect real agricultural operating conditions. Finally, long-term orchard mapping should update both geometric structures and terrain-state variables, enabling autonomous vehicles to adapt to seasonal changes, rainfall-induced soil variation, machinery ruts, and surface deformation.

## 7. Conclusions

This review summarized terrain modeling, DEM reconstruction, traversability assessment, and cost map construction methods for autonomous agricultural vehicles operating in hilly orchards. Unlike flat farmland, hilly orchards are characterized by complex terrain, dense canopy occlusion, irregular tree-row structures, narrow operating corridors, and vehicle-specific stability constraints. These conditions require navigation-oriented terrain modeling rather than purely geometric mapping.

A central conclusion of this review is that DEM quality should be evaluated not only by geometric accuracy, but also by its downstream influence on navigation safety. Upstream sensing, filtering, interpolation, and resolution-selection errors may distort DEM-derived terrain features and further affect traversability assessment, cost-map construction, and path-planning decisions. Therefore, DEM error propagation provides a useful perspective for understanding how mapping uncertainty may lead to unsafe or overly conservative navigation behavior.

Based on the reviewed studies and the synthesis presented in this review, future research should further explore uncertainty-aware terrain modeling, multimodal sensor fusion, navigation-oriented cost map representation, soil-state-aware traversability assessment, benchmark datasets for hilly orchards, and long-term map updating. These topics represent emerging priorities for improving the reliability, safety, and adaptability of autonomous agricultural vehicles in complex orchard environments.

## Figures and Tables

**Table 1:** Terrain constraints, operational risks, and traversability cost map layers for autonomous navigation in hilly orchards.

Terrain Constraint	Indicator	Operational Risk	Cost Map Layer
Longitudinal slope	Slope along vehicle heading	Climbing difficulty and wheel-slip risk	Longitudinal slope cost
Lateral slope	Slope perpendicular to vehicle heading	Rollover risk	Rollover stability cost
Surface roughness	Elevation variation or vibration index	Chassis vibration and task instability	Roughness cost
Terrain discontinuity	Breakline height, ditch width, or step height	Obstacle-crossing risk	Discontinuity/obstacle cost
Narrow corridor	Corridor width or clearance margin	Collision risk	Clearance cost
Soft soil	Soil moisture or bearing capacity	Sinkage and slip risk	Soil-state cost

**Table 2:** GNSS correction, augmentation, and optimization strategies and their relevance to DEM reconstruction in hilly orchards [42,43,44,45,46,47,48,49].

Strategy/Method	Core Function	Vertical Accuracy	Canopy Robustness	Applicability	DEM Relevance
SBAS/regional augmentation systems	Wide-area satellite corrections	Low to moderate	Limited under dense canopy	Coarse mapping	Bias reduction; limited DEM control
Local RTK base station	Real-time carrier-phase corrections	High under open-sky conditions	Sensitive to canopy blockage	Local mapping	Accurate GCPs; canopy-induced bias
CORS/network RTK	Regional RTK corrections	High when network coverage and communication are stable	Moderate; still affected by local canopy occlusion and satellite geometry	Regional mapping	Regional elevation consistency; DEM patch inconsistency risk
PPP/PPP-AR	Precise positioning without local base station	Moderate to high after convergence	Sensitive to signal interruption	Open or partially open orchards, post-processing	Base-station-free positioning; convergence limitation
PPP-RTK with multi-frequency/multi-GNSS	PPP with regional corrections	High potential accuracy; shorter convergence	Moderate; depends on signal continuity and geometry	Wide-area mapping	Vertical and DEM georeferencing stability
PPK	Post-processed kinematic correction	High after post-processing	Tolerant of real-time link loss	UAV-based mapping	IUAV trajectory refinement; strip-misalignment reduction
GNSS/INS	GNSS + inertial integration	Maintains short-term trajectory continuity	Good short-term robustness	Mobile mapping	Motion-induced DEM deformation reduction
Robust GNSS estimation algorithms	Kalman/FGO/ambiguity validation	Depends on observation quality and model design	Improves robustness under intermittent degradation	Degraded-signal environments	Vertical outlier suppression; slope/risk-layer error reduction

**Table 3:** Comparison of ALS, TLS, and MLS for DEM modeling in hilly orchards.

Platform	Ground Penetration	Point Density	Efficiency	Occlusion/Motion Sensitivity	DEM Applicability	Real-Time Use
ALS/UAV-LiDAR	Limited under dense canopy	Medium–high	High	Sensitive to canopy occlusion; low motion distortion	Regional DSM/DEM; limited under-canopy bare-earth accuracy	Limited
TLS	High local detail	Very high	Low	Sensitive to station occlusion; minimal motion distortion	Local high-accuracy DEM and validation	Low
MLS	Moderate to high along routes	High	High	Sensitive to motion distortion, synchronization, and attitude errors	Route-level DEM and navigation map construction	High

**Table 4:** Vision-based surface reconstruction and semantic mapping under different orchard scenarios.

Researcher	Data Type/Method	Scenario	Semantic Output	Scenario Limitation & DEM Applicability	Ref.
Matsuura et al.	UAV imagery, RTK-GNSS, photogrammetry, DSM reconstruction	Farmland crop-height mapping	Limited semantic output	DSM/crop-height estimation; limited bare-earth DEM extraction	[71]
Yandun Narváez et al.	ToF depth data, terrain classification	Agricultural terrain classification	Terrain class labels	Local depth/traversability assessment; illumination/range sensitivity	[72]
Bing et al.	UAV multi-view imagery, SfM, dense matching	Orchard 3D reconstruction	Limited object-level semantics	Orchard DSM/spatial modeling; limited bare-earth DEM reliability	[73]
Zhang et al.	UAV remote-sensing imagery, photogrammetry, visual modeling	Orchard perception and mapping review	Task-dependent semantic labels	UAV DEM/DSM mapping; canopy/overlap/illumination/season sensitivity	[74]
Azizi et al.	RGB agricultural scene images, semantic segmentation	Agricultural scene segmentation	Ground/non-ground or object labels	Ground recognition/DEM preprocessing; limited cross-scene generalization	[75]
Chiu et al.	UAV aerial imagery, pixel-level annotation	Agricultural semantic segmentation dataset	Crop, weed, water, road, building, and other labels	Semantic mapping dataset; ground/non-ground discrimination support	[76]

**Table 5:** Comparison of ground point filtering methods for DEM reconstruction in hilly orchards.

Filtering Method	Filtering Effect	Advantages	Limitations Under Dense Vegetation and Steep Slopes	Applicable Conditions
Elevation thresholding	Height-based ground/non-ground separation	Simple, computationally efficient, easy to implement	Slope sensitivity, ground misclassification	Smooth terrain; sparse low vegetation
Morphological filtering	Removes non-ground objects using moving windows	Effective for small vegetation and local noise	Window-size sensitivity, smoothing artifacts	Moderate terrain complexity; continuous surfaces
Progressive TIN densification	Builds ground TIN and adds candidate points progressively	Preserves terrain continuity; adapts to elevation changes	Seed-point dependence, terrain sensitivity	Hilly terrain with sufficient ground returns
Grid-based filtering	Extracts ground points using grid elevation statistics	Efficient; compatible with raster DEMs and cost maps	Grid-size dependence, small-feature loss	Real-time mapping; raster-based navigation
Geometric feature methods	Uses normals, curvature, slope, or PCA features	Distinguishes terrain from vegetation structures	Noise/density/spacing sensitivity	Dense LiDAR data with clear geometry
Learning-based segmentation	Learns ground/non-ground labels from data	Handles complex vegetation and irregular terrain	Label dependence, limited generalization	Data-rich scenarios with representative samples

**Table 6:** Interpolation artifacts and navigation impacts in DEM generation.

Interpolation Method	Typical Artifact/Limitation	Effect on DEM Features	Navigation Impact	Mitigation Strategies	Ref.
IDW	Bull’s-eye effect under sparse or uneven points	Artificial local highs/lows; distorted slope and roughness	False roughness or obstacle cost	Adaptive search radius; density control; artifact filtering	[104]
Spline	Oversmoothing or overshoot near sharp terrain changes	Weakened ditches, steps, and breaklines	Missed discontinuities; underestimated obstacle-crossing risk	Breakline constraints; local validation; multi-scale checking	[104]
Kriging	Variogram-dependent bias; parameter sensitivity	Oversmoothed or locally biased surface	Unstable risk boundaries; unreliable uncertainty estimates	Variogram calibration; cross-validation; uncertainty mapping	[104]
TIN	Triangular facet artifacts in sparse regions	Planar facets; unrealistic slope changes	Cost map discontinuities; biased local path preference	Density-aware triangulation; breakline preservation; validated raster smoothing	[105]
Learning-based filling	Overconfident reconstruction in occluded areas	Plausible but unobserved surfaces	Overconfident planning in uncertain regions	Uncertainty estimation; field validation; physical-constraint fusion	[106]

**Table 7:** DEM quality assessment, error propagation, and their navigational impacts.

Researcher	Research Object	Research Method	Research Objective	Ref.
Bui and Glennie	DEM uncertainty assessment	Quantified LiDAR raster DEM uncertainty, analyzing the impact of terrain roughness and point cloud density	Expand from elevation accuracy evaluation to uncertainty representation	[108]
Bei et al.	Impact of DEM errors on terrain features	Compared DEM accuracy and roughness variations under different point cloud densities and interpolation methods	Demonstrate the propagation of DEM errors into derived features like roughness	[109]
Zhang et al.	Mapping terrain features to risk costs	Generated risk cost maps based on DEM feature extraction and learning from demonstration	Establish the correlation between geometric errors and risk identification	[110]

**Table 8:** DEM-derived terrain risk factors and possible cost representations.

Risk Factor	Typical Indicator	Cost Type	Planner-Level Effect
Longitudinal slope	Slope along vehicle heading	Threshold, continuous penalty, fuzzy membership	Uphill/downhill route penalty
Lateral slope	Slope perpendicular to heading	Stability margin, rollover-risk function, threshold	Rollover-risk avoidance
Surface roughness	Local elevation variance, height residuals, vibration-related index	Continuous roughness cost, vibration penalty	Smooth-path preference
Terrain discontinuity	Breakline height, ditch width, step height	Hard constraint, obstacle-crossing threshold, high cost	Impassable-region exclusion
Obstacle boundary	Height difference, occupancy boundary, inflated obstacle region	Occupancy cost, distance transform, inflation layer	Clearance maintenance
Curvature/local relief	Curvature, local relief, terrain gradient variation	Continuous terrain-complexity cost	Maneuverability improvement

**Table 9:** Summary of research on normalization and fusion of multimodal cost factors.

Researcher	Research Object	Research Method	Research Objective	Ref.
Fan et al.	Traversability evaluation and hazard/safety map reconstruction in complex terrain	Dual-branch semantic segmentation and fuzzy logic evaluation	Improve representation of fuzzy boundaries and enhance path cost robustness	[119]
Zhang et al.	Adaptive cost modeling under vehicle–terrain interaction	Vehicle–terrain interaction model and fuzzy inference weighting	Enhance path planning robustness in complex terrain	[120]
Zhou et al.	Multimodal perception traversability mapping	Visual/LiDAR traversable area extraction and Bayesian fusion	Improve traversability estimation accuracy and stability	[121]
Endo et al.	Risk-aware path planning for heterogeneous surfaces	Multi-model probabilistic fusion and risk cost construction	Strengthen planning reliability under surface uncertainty	[122]
Feng et al.	Terrain–vehicle dynamics coupling for risk modeling	Constraint-aware motion planning and joint traversability assessment	Improve path feasibility and safety in complex scenarios	[123]
Wang et al.	Multi-layer traversability cost map construction	Multi-factor fusion and piecewise linear cost modeling	Achieve unified cost representation for heterogeneous terrain factors	[124]

## Data Availability

No new data were created or analyzed in this study. Data sharing is not applicable to this article.

## References

[1] Jiang L., Xu B., Husnain N., Wang Q. (2025). Overview of Agricultural Machinery Automation Technology for Sustainable Agriculture. Agronomy.

[2] Taha M.F., Mao H., Mousa S., Zhou L., Wang Y., Elmasry G., Al-Rejaie S., Elwakeel A.E., Wei Y., Qiu Z. (2024). Deep Learning-Enabled Dynamic Model for Nutrient Status Detection of Aquaponically Grown Plants. Agronomy.

[3] Li C., Cheng S., Aziz F., Yang J., Yu S. (2026). Agricultural Science: A CiteSpace-Based Bibliometric Analysis of Global and Chinese Research. J. Sci. Food Agric..

[4] Li H., Chen L., Zhang Z. (2023). A Study on the Utilization Rate and Influencing Factors of Small Agricultural Machinery: Evidence from 10 Hilly and Mountainous Provinces in China. Agriculture.

[5] Wang R., Zhang K., Ding R., Jiang Y., Jiang Y. (2025). A Novel Hydraulic Interconnection Design and Sliding Mode Synchronization Control of Leveling System for Crawler Work Machine. Agriculture.

[6] Wang H. (2023). Research on the Scheduling of Plant Protection UAV Spraying Operations in Hilly Orchards. Master’s Thesis.

[7] Li Z., Zhu X., Yao S., Yue Y., García-Fernández Á.F., Lim E.G., Levers A. (2023). A Large Scale Digital Elevation Model Super-Resolution Transformer. Int. J. Appl. Earth Obs. Geoinf..

[8] Polidori L., El Hage M. (2020). Digital Elevation Model Quality Assessment Methods: A Critical Review. Remote Sens..

[9] DeJong T.M. (2019). Opportunities and Challenges in Fruit Tree and Orchard Modelling. Eur. J. Hortic. Sci..

[10] Wijayathunga L., Rassau A., Chai D. (2023). Challenges and Solutions for Autonomous Ground Robot Scene Understanding and Navigation in Unstructured Outdoor Environments: A Review. Appl. Sci..

[11] Jiang Y., Wang R., Ding R., Sun Z., Jiang Y., Liu W. (2025). Research Review of Agricultural Machinery Power Chassis in Hilly and Mountainous Areas. Agriculture.

[12] Zheng Y., Jiang S., Chen B., Lyu H., Wan C., Kang F. (2020). Review on Technology and Equipment of Mechanization in Hilly Orchard. Trans. Chin. Soc. Agric. Mach..

[13] Wang M., Xu J., Zhang J., Cui Y. (2024). An Autonomous Navigation Method for Orchard Rows Based on a Combination of an Improved A-Star Algorithm and SVR. Precis. Agric..

[14] Ramsey J., Brothers R., Hernandez J. (2024). Creation of a Ground Slope Mapping Methodology Within the Robotic Technology Kernel for Improved Navigation Performance.

[15] Ding R., Qi X., Chen X., Mei Y., Li A., Wang R., Guo Z. (2025). Research on the Design of an Omnidirectional Leveling System and Adaptive Sliding Mode Control for Tracked Agricultural Chassis in Hilly and Mountainous Terrain. Agriculture.

[16] Bao X., Ma Z., Ma X., Li Y., Ren M., Li S. (2024). Design and Experiment of Citrus Picking Robot in Hilly Orchard Natural Environment. Trans. Chin. Soc. Agric. Mach..

[17] Puri D., Vita L., Gattamelata D., Tulliani V. (2025). Roll/Tip-Over Risk Analysis of Agricultural Self-Propelled Machines Using Airborne LiDAR Data: GIS-Based Approach. Machines.

[18] Li Y., Qi B., Bao E., Tang Z., Lian Y., Sun M. (2025). Design and Analysis of Sowing Depth Detection and Control Device for Multi-Row Wheat Seeders Adapted to Different Terrain Variations. Agriculture.

[19] Chen K., Li T., Yan T., Xie F., Feng Q., Zhu Q., Zhao C. (2022). A Soft Gripper Design for Apple Harvesting with Force Feedback and Fruit Slip Detection. Agriculture.

[20] Wu M., Liu S., Li Z., Ou M., Dai S., Dong X., Wang X., Jiang L., Jia W. (2025). A Review of Intelligent Orchard Sprayer Technologies: Perception, Control, and System Integration. Horticulturae.

[21] Tibermacine A., Tibermacine I.E., Akrour D., Rabehi A., Habib M. (2026). Autonomous Navigation in Unstructured Outdoor Environments Using Semantic Segmentation Guided Reinforcement Learning. Sci. Rep..

[22] Yue Z., Sun C., Zhang X., Tang C., Gao Y., Li K. (2025). A Semantic Segmentation-Based GNSS Signal Occlusion Detection and Optimization Method. Remote Sens..

[23] Sun C., Sun J., Ding S., Li Q., Ma L. (2025). Path Tracking Control in Autonomous Agricultural Vehicles: A Systematic Survey of Models, Methods, and Challenges. Agriculture.

[24] Wang Z., Zhu X., Wang F. (2025). Effects of Longitudinal and Transverse Travel Direction on the Hydraulic Performance of Sprinkler Machines on Sloping Terrain. Agriculture.

[25] Sun J., Meng X., Zeng L., Zheng H., Ying J., Zhang H., Xu G. (2025). Design and Performance Test of Experimental Platform for Omnidirectional Control of Agricultural Chassis Center of Gravity in Hilly and Mountainous Areas. Trans. Chin. Soc. Agric. Mach..

[26] Mu X., Yang F., Duan L., Liu Z., Song Z., Li Z., Guan S. (2024). Research Advances and Development Trend of Mountainous Tractor Leveling and Anti-Rollover System. Smart Agric..

[27] Liu H., Yan S., Shen Y., Li C., Zhang Y., Hussain F. (2021). Model Predictive Control System Based on Direct Yaw Moment Control for 4WID Self-Steering Agriculture Vehicle. Int. J. Agric. Biol. Eng..

[28] Chen P., Liu S., Xu J., Liu M. (2025). Stability Control of a Wheel-Legged Mobile Platform Used in Hilly Orchards. Biosyst. Eng..

[29] Zhang Y., Liu H., Shen Y., He S., Wang H., Shen Y. (2025). A Systematic Review of Modeling and Control Approaches for Path Tracking in Unmanned Agricultural Ground Vehicles. Agronomy.

[30] Cui L., Xue X., Le F., Mao H., Ding S. (2019). Design and Experiment of Electro Hydraulic Active Suspension for Controlling the Rolling Motion of Spray Boom. Int. J. Agric. Biol. Eng..

[31] Ding R., Qi X., Meng X., Chen X., Zhang L., Mei Y., Li A., Ye Q. (2025). A Review on the Chassis Configurations and Key Technologies of Agricultural Robots. Agriculture.

[32] Liu C., Lin H., Li Y., Gong L., Miao Z. (2020). Analysis on Status and Development Trend of Intelligent Control Technology for Agricultural Equipment. Trans. Chin. Soc. Agric. Mach..

[33] Mei S., Xia X., He J., Song Z., Cao G. (2025). Current Status and Key Development Priorities of Efficient Mechanized Production Equipment in Orchards. Chin. Agric. Sci. Bull..

[34] Wu H., Wang X., Chen X., Zhang Y., Zhang Y. (2025). Review on Key Technologies for Autonomous Navigation in Field Agricultural Machinery. Agriculture.

[35] Cui B., Cui X., Wei X., Zhu Y., Ma Z., Zhao Y., Liu Y. (2024). Design and Testing of a Tractor Automatic Navigation System Based on Dynamic Path Search and a Fuzzy Stanley Model. Agriculture.

[36] Nguyen N.V., Cho W. (2023). Performance Evaluation of a Typical Low-Cost Multi-Frequency Multi-GNSS Device for Positioning and Navigation in Agriculture—Part 2: Dynamic Testing. AgriEngineering.

[37] Kowalczyk W.Z., Hadas T. (2024). A Comparative Analysis of the Performance of Various GNSS Positioning Concepts Dedicated to Precision Agriculture. Rep. Geod. Geoinform..

[38] Udompant K., Ospina R., Kim Y.-J., Noguchi N. (2021). Utilization of Quasi-Zenith Satellite System for Navigation of a Robot Combine Harvester. Agronomy.

[39] Jiang Y., Ding W., Gao Y., Gao Y. (2022). A New Partial Ambiguity Resolution Method Based on Modified Solution Separation and GNSS Epoch-Differencing. J. Geod..

[40] Ma W., Dai W., Yu W., Fang Y., Zhao X., Guo Y. (2025). Kalman Filtering-Enhanced Factor Graph Optimization for GNSS RTK in Complex Environment. IEEE Sens. J..

[41] Wang X., Li X., Shen Z., Li X., Zhou Y., Chang H. (2023). Factor Graph Optimization-Based Multi-GNSS Real-Time Kinematic System for Robust and Precise Positioning in Urban Canyons. GPS Solut..

[42] Krasuski K., Wierzbicki D., Bakuła M. (2021). Improvement of UAV Positioning Performance Based on EGNOS+SDCM Solution. Remote Sens..

[43] Niu Z., Xia H., Tao P., Ke T. (2024). Accuracy Assessment of UAV Photogrammetry System with RTK Measurements for Direct Georeferencing. ISPRS Ann. Photogramm. Remote Sens. Spat. Inf. Sci..

[44] Pipitone C., Maltese A., Lo Brutto M., Dardanelli G. (2023). A Review of Selected Applications of GNSS CORS and Related Experiences at the University of Palermo (Italy). Remote Sens..

[45] Erol B., Turan E., Erol S., Kuçak R.A. (2024). Comparative Performance Analysis of Precise Point Positioning Technique in the UAV-Based Mapping. Measurement.

[46] Li X., Huang J., Li X., Shen Z., Han J., Li L., Wang B. (2022). Review of PPP–RTK: Achievements, Challenges, and Opportunities. Satell. Navig..

[47] Martínez-Carricondo P., Agüera-Vega F., Carvajal-Ramírez F. (2023). Accuracy Assessment of RTK/PPK UAV-Photogrammetry Projects Using Differential Corrections from Multiple GNSS Fixed Base Stations. Geocarto Int..

[48] Boguspayev N., Akhmedov D., Raskaliyev A., Kim A., Sukhenko A. (2023). A Comprehensive Review of GNSS/INS Integration Techniques for Land and Air Vehicle Applications. Appl. Sci..

[49] Zou Z., Wang G., Li Z., Zhai R., Li Y. (2024). MFO-Fusion: A Multi-Frame Residual-Based Factor Graph Optimization for GNSS/INS/LiDAR Fusion in Challenging GNSS Environments. Remote Sens..

[50] Sun Y., Luo Y., Zhang Q., Xu L., Wang L., Zhang P. (2022). Estimation of Crop Height Distribution for Mature Rice Based on a Moving Surface and 3D Point Cloud Elevation. Agronomy.

[51] Rivera G., Porras R., Florencia R., Sánchez-Solís J.P. (2023). LiDAR Applications in Precision Agriculture for Cultivating Crops: A Review of Recent Advances. Comput. Electron. Agric..

[52] Yu S., Zhu J., Zhou J., Cheng J., Bian X., Shen J., Wang P. (2022). Key Technology Progress of Plant-Protection UAVs Applied to Mountain Orchards: A Review. Agronomy.

[53] Manish R., Habib A. (2024). In-Situ Calibration and Trajectory Enhancement of UAV LiDAR Systems for Mapping Mechanized Agricultural Fields. IEEE J. Sel. Top. Appl. Earth Obs. Remote Sens..

[54] Fareed N., Das A.K., Flores J.P. (2023). Quality Control and Crop Characterization Framework Using Unmanned Aerial Systems (UAS) LiDAR Point Clouds over Agricultural Fields. Proceedings of the 2023 ASABE Annual International Meeting, Omaha, NE, USA, 9–12 July 2023.

[55] Khan N.H.R., Kumar S.V. (2024). Terrestrial LiDAR Derived 3D Point Cloud Model, Digital Elevation Model (DEM) and Hillshade Map for Identification and Evaluation of Pavement Distresses. Results Eng..

[56] Xu J., Liu H., Shen Y., Zeng X., Zheng X. (2024). Individual Nursery Trees Classification and Segmentation Using a Point Cloud-Based Neural Network with Dense Connection Pattern. Sci. Hortic..

[57] Qu J., Qiu Z., Li L., Guo K., Li D. (2024). Map Construction and Positioning Method for LiDAR SLAM-Based Navigation of an Agricultural Field Inspection Robot. Agronomy.

[58] Hong Y., Ma R., Li C., Shao C., Huang J., Zeng Y., Chen Y. (2024). Three-Dimensional Localization and Mapping of Multiagricultural Scenes via Hierarchically-Coupled LiDAR-Inertial Odometry. Comput. Electron. Agric..

[59] Teng H., Wang Y., Chatziparaschis D., Karydis K. (2025). Adaptive LiDAR Odometry and Mapping for Autonomous Agricultural Mobile Robots in Unmanned Farms. Comput. Electron. Agric..

[60] Rakun J., Duchoň F., Lepej P. (2024). Spatial LiDAR Odometry and Mapping for Complex Agricultural Environments: Spatial Field-LOAM. Biosyst. Eng..

[61] Ji W., Gao X., Xu B., Pan Y., Zhang Z., Zhao D. (2021). Apple Target Recognition Method in Complex Environment Based on Improved YOLOv4. J. Food Process Eng..

[62] Lv R., Hu J., Zhang T., Chen X., Liu W. (2025). Crop-Free-Ridge Navigation Line Recognition Based on the Lightweight Structure Improvement of YOLOv8. Agriculture.

[63] Wang H., Gu J., Wang M. (2023). A Review on the Application of Computer Vision and Machine Learning in the Tea Industry. Front. Sustain. Food Syst..

[64] Ma Z., Yang S., Li J., Qi J. (2024). Research on SLAM Localization Algorithm for Orchard Dynamic Vision Based on YOLOD-SLAM2. Agriculture.

[65] Yang T., Du X., Zhang B., Wang X., Zhang Z., Wu C. (2025). Coverage Path Planning Based on Region Segmentation and Path Orientation Optimization. Agriculture.

[66] Liu W., Hu J., Liu J., Yue R., Zhang T., Yao M., Li J. (2024). Method for the Navigation Line Recognition of the Ridge without Crops via Machine Vision. Int. J. Agric. Biol. Eng..

[67] Xu J., Liu H., Shen Y. (2025). Image and Point Cloud-Based Neural Network Models and Applications in Agricultural Nursery Plant Protection Tasks. Agronomy.

[68] Syed T.N., Zhou J., Lakhiar I.A., Marinello F., Gemechu T.T., Rottok L.T., Jiang Z. (2025). Enhancing Autonomous Orchard Navigation: A Real-Time Convolutional Neural Network-Based Obstacle Classification System for Distinguishing ‘Real’ and ‘Fake’ Obstacles in Agricultural Robotics. Agriculture.

[69] Chen J., Song J., Guan Z., Lian Y. (2021). Measurement of the Distance from Grain Divider to Harvesting Boundary Based on Dynamic Regions of Interest. Int. J. Agric. Biol. Eng..

[70] Luo Y., Wei L., Xu L., Zhang Q., Liu J., Cai Q., Zhang W. (2022). Stereo-Vision-Based Multi-Crop Harvesting Edge Detection for Precise Automatic Steering of Combine Harvester. Biosyst. Eng..

[71] Matsuura Y., Zhang H., Nakao K., Chang Q., Firmansyah I., Shimizu H. (2023). High-Precision Plant Height Measurement by Drone with RTK-GNSS and Single Camera for Real-Time Processing. Sci. Rep..

[72] Narváez F.Y., Gregorio E., Escolà A., Torres-Torriti M., Cheein F.A., Rosell-Polo J.R. (2018). Terrain Classification Using ToF Sensors for the Enhancement of Agricultural Machinery Traversability. J. Terramech..

[73] Bing Q., Zhang R., Zhang L., Li L., Chen L. (2025). UAV-SfM Photogrammetry for Canopy Characterization Toward Unmanned Aerial Spraying Systems Precision Pesticide Application in an Orchard. Drones.

[74] Zhang C., Valente J., Kooistra L., Guo L., Wang W. (2021). Orchard Management with Small Unmanned Aerial Vehicles: A Survey of Sensing and Analysis Approaches. Precis. Agric..

[75] Azizi A., Abbaspour-Gilandeh Y., Vannier E., Dusséaux R., Mseri-Gundoshmian T., Moghaddam H.A. (2020). Semantic Segmentation: A Modern Approach for Identifying Soil Clods in Precision Farming. Biosyst. Eng..

[76] Chiu M.T., Xu X., Wei Y., Huang Z., Schwing A.G., Brunner R. (2020). Agriculture-Vision: A Large Aerial Image Database for Agricultural Pattern Analysis. Proceedings of the IEEE/CVF Conference on Computer Vision and Pattern Recognition (CVPR), Seattle, WA, USA, 13–19 June 2020.

[77] Zhang Y., Zhang B., Shen C., Liu H., Huang J., Tian K., Tang Z. (2024). Review of the Field Environmental Sensing Methods Based on Multi-Sensor Information Fusion Technology. Int. J. Agric. Biol. Eng..

[78] Shen Y., Xiao X., Liu H., Zhang X. (2023). Real-Time Localization and Mapping Method for Agricultural Robot in Orchards Based on LiDAR/IMU Tight-Coupling. Trans. Chin. Soc. Agric. Mach..

[79] Chen Z., Yin J., Farhan S.M., Liu L., Zhang D., Zhou M., Cheng J. (2026). A Comprehensive Review of Obstacle Avoidance for Autonomous Agricultural Machinery in Multi-Operational Environment. Artif. Intell. Agric..

[80] Ma Z., Wang X., Chen X., Hu B., Li J. (2025). Advances in Crop Row Detection for Agricultural Robots: Methods, Performance Indicators, and Scene Adaptability. Agriculture.

[81] Liu H., Huang Y., Shen Y., Shen Y., Wu R. (2026). Synchronously Locating and Mapping of Orchard Spraying Robots Using Trunk Features and Loop Closure Optimization. Trans. Chin. Soc. Agric. Eng..

[82] Guan X., Ge H., Nie S., Ding Y. (2025). Research on Agricultural Autonomous Positioning and Navigation System Based on LIO-SAM and Apriltag Fusion. Agronomy.

[83] Jiang S., Qi P., Han L., Liu L., Li Y., Huang Z., Liu Y., He X. (2024). Navigation System for Orchard Spraying Robot Based on 3D LiDAR SLAM with NDT-ICP Point Cloud Registration. Comput. Electron. Agric..

[84] Xia Y., Lei X., Pan J., Chen L., Zhang Z., Lyu X. (2023). Research on Orchard Navigation Method Based on Fusion of 3D SLAM and Point Cloud Positioning. Front. Plant Sci..

[85] Yu Y., Li Z., Dai B., Pan J., Xu L. (2025). High-Precision Mapping and Real-Time Localization for Agricultural Machinery Sheds and Farm Access Roads Environments. Agriculture.

[86] Choi S., Han X., Chang E., Jeong H. (2025). LiDAR-IMU Sensor Fusion-Based SLAM for Enhanced Autonomous Navigation in Orchards. Agriculture.

[87] Duan Y. (2023). Research on Autonomous Navigation Methods of Mobile Robots in Forests and Orchards Based on LiDAR. Master’s Thesis.

[88] Zhao Z., Zhang Y., Long L., Lu Z., Shi J. (2022). Efficient and Adaptive LiDAR–Visual–Inertial Odometry for Agricultural Unmanned Ground Vehicle. Int. J. Adv. Robot. Syst..

[89] Sun N., Qiu Q., Li T., Ru M., Ji C., Feng Q., Zhao C. (2024). GNSS/LiDAR/IMU Fusion Odometry Based on Tightly-Coupled Nonlinear Observer in Orchard. Remote Sens..

[90] Zhang S., Xue X., Chen C., Sun Z., Sun T. (2019). Development of a Low-Cost Quadrotor UAV Based on ADRC for Agricultural Remote Sensing. Int. J. Agric. Biol. Eng..

[91] Khan Z., Shen Y., Liu H. (2025). Object Detection in Agriculture: A Comprehensive Review of Methods, Applications, Challenges, and Future Directions. Agriculture.

[92] Chen J., Zhu F., Guan Z., Zhu Y., Shi H., Cheng K. (2024). Development of a Combined Harvester Navigation Control System Based on Visual Simultaneous Localization and Mapping-Inertial Guidance Fusion. J. Agric. Eng..

[93] Han C., Wu W., Luo X., Li J. (2023). Visual Navigation and Obstacle Avoidance Control for Agricultural Robots via LiDAR and Camera. Remote Sens..

[94] Liu H., Gu H., Wang Y., Zhong T., Tian T., Geng C. (2026). A GNSS–Vision Integrated Autonomous Navigation System for Trellis Orchard Transportation Robots. Appl. Inform..

[95] Fanta-Jende P., Steininger D., Kern A., Widhalm V., Baca J.G.A., Sulzer W. (2023). Semantic Real-Time Mapping with UAVs. PFG–J. Photogramm. Remote Sens. Geoinf. Sci..

[96] Cai Y., Cui B., Lu Z., Xie Y. (2026). Extraction of Points and Efficiency Analysis of Fruit Orchard Operation Platform Based on Grid Division. J. Chin. Agric. Mech..

[97] Li H., Huang K., Sun Y., Lei X., Yuan Q., Lv X. (2025). An Autonomous Navigation Method for Orchard Mobile Robots Based on Octree 3D Point Cloud Optimization. Front. Plant Sci..

[98] Steinke N., Goehring D., Rojas R. (2024). GroundGrid: LiDAR Point Cloud Ground Segmentation and Terrain Estimation. IEEE Robot. Autom. Lett..

[99] Wu B., Zhou X., Zhao J., Zhang W., Zheng G. (2025). A Data-Driven Morphological Filtering Algorithm for Digital Terrain Model Generation from Airborne LiDAR Data. ISPRS Open J. Photogramm. Remote Sens..

[100] Santo A., Heredia E., Viegas C., Valiente D., Gil A. (2025). Ground Segmentation for LiDAR Point Clouds in Structured and Unstructured Environments Using a Hybrid Neural–Geometric Approach. Technologies.

[101] Habib M., Alzubi Y., Malkawi A., Awwad M., Albattah M., Abu-Hudrouss A. (2020). Impact of Interpolation Techniques on the Accuracy of Large-Scale Digital Elevation Model. Open Geosci..

[102] Li Z., Wang K., Ma H., Wu Y. (2018). An Adjusted Inverse Distance Weighted Spatial Interpolation Method. Proceedings of the 2018 3rd International Conference on Communications, Information Management and Network Security (CIMNS 2018).

[103] Li L., Nearing M.A., Nichols M.H., Polyakov V.O., Guertin D.P., Cavanaugh M.L. (2020). The Effects of DEM Interpolation on Quantifying Soil Surface Roughness Using Terrestrial LiDAR. Soil Tillage Res..

[104] Adedapo S.M., Zurqani H.A. (2024). Evaluating the Performance of Various Interpolation Techniques on Digital Elevation Models in Highly Dense Forest Vegetation Environment. Ecol. Inform..

[105] He Z., Gan S., Yuan X. (2026). A Terrain-Constrained TIN Approach for High-Precision DEM Reconstruction Using UAV Point Clouds. J. Imaging.

[106] Stölzle M., Miki T., Gerdes L., Azkarate M., Hutter M. (2022). Reconstructing Occluded Elevation Information in Terrain Maps with Self-Supervised Learning. IEEE Robot. Autom. Lett..

[107] Chen W., Yang J., Zhang S., Wei X., Liu C., Zhou X., Sun L., Wang F., Wang A. (2025). Variable Scale Operational Path Planning for Land Levelling Based on the Improved Ant Colony Optimization Algorithm. Sci. Rep..

[108] Bui L.K., Glennie C.L. (2023). Estimation of LiDAR-Based Gridded DEM Uncertainty with Varying Terrain Roughness and Point Density. ISPRS Open J. Photogramm. Remote Sens..

[109] Bei Y., Chen C., Wang X., Sun Y., He Q., Li K. (2023). Effects of Airborne LiDAR Point Cloud Density and Interpolation Methods on the Accuracy of DEM and Surface Roughness. J. Geo-Inf. Sci..

[110] Zhang B., Li G., Zhang J., Bai X. (2024). A Reliable Traversability Learning Method Based on Human-Demonstrated Risk Cost Mapping for Mobile Robots over Uneven Terrain. Eng. Appl. Artif. Intell..

[111] Shu Y., Dong L., Liu J., Liu C., Wei W. (2025). Overview of Terrain Traversability Evaluation for Autonomous Robots. J. Field Robot..

[112] Cao G., Zhang B., Li Y., Zhou J., Yue W., He X. (2025). Environmental Mapping and Path Planning for Robots in Orchard Based on Traversability Analysis, Improved LeGO-LOAM and RRT* Algorithms. Comput. Electron. Agric..

[113] Su Z., Zou W., Zhai C., Tan H., Yang S., Qin X. (2024). Design of an Autonomous Orchard Navigation System Based on Multi-Sensor Fusion. Agronomy.

[114] Wang W., Qin J., Huang D., Zhang F., Liu Z., Wang Z., Yang F. (2024). Integrated Navigation Method for Orchard-Dosing Robot Based on LiDAR/IMU/GNSS. Agronomy.

[115] Jiang A., Ahamed T. (2023). Navigation of an Autonomous Spraying Robot for Orchard Operations Using LiDAR for Tree Trunk Detection. Sensors.

[116] Rauf O., Ning Y., Ming C., Haoxiang M. (2024). Evaluation of Ground Pressure, Bearing Capacity, and Sinkage in Rigid-Flexible Tracked Vehicles on Characterized Terrain in Laboratory Conditions. Sensors.

[117] Shi Y., Liu J., Huang D., Xu M., Zhai S., Zhang W., Jiang P. (2023). Prediction and Experimental Study of Tire Slip Rate Based on Chassis Sinkage Amount. Agriculture.

[118] Pan Y., Hu K., Cao H., Kang H., Wang X. (2024). A Novel Perception and Semantic Mapping Method for Robot Autonomy in Orchards. Comput. Electron. Agric..

[119] Fan L., Yuan J., Zha K. (2025). TerSeg: A Dual-Branch Semantic Segmentation Network for Mars Terrain and Autonomous Path Planning. Expert Syst. Appl..

[120] Zhang H., Zhao Q., Wu Y., Jiang D., Chen X., Liang X., Sun Y. (2025). Integration of Vehicle–Terrain Interaction and Fuzzy Cost Adaptation for Robust Path Planning. Sensors.

[121] Zhou L., Wang J., Lin S., Chen Z. (2022). Terrain Traversability Mapping Based on LiDAR and Camera Fusion. Proceedings of the 2022 8th International Conference on Automation, Robotics and Applications (ICARA), Prague, Czech Republic, 18–20 February 2022.

[122] Endo M., Taniai T., Yonetani R., Ishigami G. (2023). Risk-Aware Path Planning via Probabilistic Fusion of Traversability Prediction for Planetary Rovers on Heterogeneous Terrains. Proceedings of the IEEE International Conference on Robotics and Automation (ICRA), London, UK, 29 May–2 June 2023.

[123] Feng C., Wang J., Wang Q., Shen Y., Wei Y., Zhang S., Zhang S., Zhang Q. (2025). Constraint-Aware Motion Planning for Vehicles with Terrain Traversability Assessment and Optimization in Construction Scenarios. Adv. Eng. Inform..

[124] Wang N., Li X., Suo Z., Fan J., Wang J., Xie D. (2024). Traversability Analysis and Path Planning for Autonomous Wheeled Vehicles on Rigid Terrains. Drones.

[125] Ji Y., Liu Y., Xie G., Ren Q., Liu B., Ren L. (2025). NNPP: A Learning-Based Heuristic Model for Accelerating Optimal Path Planning on Uneven Terrain. Robot. Auton. Syst..

[126] Shen Y., Shen Y., Zhang Y., Huo C., Shen Z., Su W., Liu H. (2025). Research Progress on Path Planning and Tracking Control Methods for Orchard Mobile Robots in Complex Scenarios. Agriculture.

[127] Carvalho A.E., Portugal D., Peixoto P. (2025). On Terrain Traversability Analysis in Unstructured Environments: Recent Advances in Forest Applications. Intell. Serv. Robot..

[128] Liu H., Xue M., Guan H., Tao J., Chen H. (2024). Path Planning Algorithm Based on Layered 2.5D Map for Unmanned Tracked Vehicle. Trans. Beijing Inst. Technol..

[129] Sun J., Zhang X., Zhang S., Zhao M., Hu S., Zhang S., Zou Y., He J. (2026). Unstructured Road Reference Line Generation Algorithm Based on Cost Map. Automot. Eng..

[130] Chen M., Tan Y., Yu L., Liu Y., Wang H., Zhang X. (2025). Path Planning for Tracked Vehicles Based on Improved Hybrid A* Algorithm. Trans. Beijing Inst. Technol..

[131] Zang Z., Gong X., Zhang X. (2025). Coordinated Path Planning for Autonomous Ground Vehicles in Off-Road Environments with 3D Rigid Terrain and Obstacles. Knowl. Based Syst..

[132] Zhu Q., Sun Z., Xia S., Liu G., Ma K., Pei L., Gong Z. (2024). Learning-Based Traversability Costmap for Autonomous Off-Road Navigation. Proceedings of the China Intelligent Robotics Annual Conference, Singapore, 2024.

[133] Gholami A., Ramirez-Serrano A. (2025). Terrain Traversability via Sensed Data for Robots Operating Inside Heterogeneous, Highly Unstructured Spaces. Sensors.

[134] Jerome O., Bassey C., Okeme C., Maloletov A., Kulathunga G. (2025). MeshNav3D: Software for Visualizing and Benchmarking Uneven Terrain Planning Algorithms. J. Open Res. Softw..

[135] Carvalho A.E., Ferreira J.F., Portugal D. (2024). 3D Traversability Analysis and Path Planning Based on Mechanical Effort for UGVs in Forest Environments. Robot. Auton. Syst..

[136] Esfandiyar I., Belter D. (2025). Mapping the Unseen Ground: A Survey on Dense Mapping and Traversability Methods for Agricultural and Forestry Robots. IEEE Access.

[137] Vecchio G., Palazzo S., Guastella D.C., Giordano D., Spampinato C. (2024). Terrain Traversability Prediction through Self-Supervised Learning and Unsupervised Domain Adaptation on Synthetic Data. Auton. Robot..

